# Book Reviews

**Published:** 1898-10

**Authors:** 


### Book Reviews.

Wounds in War; the Mechanism of Their Production and Their Treatment. By Surgeon-Colonel W. F. Stevenson, Professor of Military Surgery, Army Medical School, Netley, England. One volume of 588 pages, 8vo, profusely illustrated. New York: William Wood & Co. 1898.

The literature of military surgery, so-called, has been increased very much by the recent war with Spain. The fact is patent to every practical surgeon that military surgery differs in no wise from civil surgery, except as to environment. Sometimes the military surgeon must temporise, when in civil life he would be radical, and vice versa. But, given a gunshot wound by a given missile, and the principles of treatment are the same, whether the victim be a soldier or a civilian.

The work before us is a practical exposé of the present status of the treatment of wounds in war. It also takes up the methods of their production through the medium of modern firearms and projectiles. Contrary to the popular belief, immediately fatal wounds on the battlefield are very rare, and when they do occur they are generally due to primary hemorrhage. This, however, is less a cause for apprehension since the now quite general use of the "ready aid" packet, with which each officer and man is supplied. Many lives were saved through this means during the Santiago campaign.

The prevention of sepsis is another important factor in the preservation of the lives of soldiers. This also can be largely done by the intelligent use of the ready aid packet. Beyond everything gunshot wounds should next to never be subjected to the probe. This now almost obsolete method has been a chief cause of wound infection and should only be exercised under the most exceptional conditions and with the most perfect antiseptic care.

This work is full of practical teaching on this and other points connected with the immediate and remote care of wounds in war.

The final chapter contains the agreement between the civilised powers arrived at during the Geneva convention, the articles of which were signed August 22, 1864. Every military officer, and especially every officer of the medical staff, should make himself familiar with the work of this convention.

The question of outside aid to the medical department of the army has always been a difficult one to adjust satisfactorily. It is, however, certain that no army is ever conditioned to render immediate and sufficient aid to all the wounded after a great battle—a battle which numbers the wounded by thousands—no matter how complete its equipment. For days after such a battle volunteer aid societies are of the greatest service in performing duties that cannot possibly be undertaken by the medical staff.

This book is written by an experienced medical officer of high rank, is most interesting in style, instructive in material, and should be found as a part of the equipment of every military medical officer from the Surgeon General to the acting assistant-surgeon.

---

TWENTIETH CENTURY PRACTICE. An International Encyclopedia of Modern Medical Science. By leading authorities of Europe and America. Edited by THOMAS L. STEDMAN, M. D., New York City. In twenty volumes. Vol. XV., Infectious Diseases. New York: William Wood & Co. 1898.

This volume, like its predecessor, is devoted to the treatment of infectious diseases, the first being influenza. In view of the widespread nature of this disease, reaching as it does into all countries and climes, this article by Ditman Finkler will prove of especial interest at this time. He gives a brief chronologic reference to its epidemiology from 1173 to the present and also treats of the disease from the bacteriological standpoint. In this latter part of the subject he is exhaustive if not conclusive.

The next topic is typhus fever, which, in view of the new possessions about to come into our hands, will become of greater interest than formerly. It had almost become an extinct disease in the United States, but in Latin-America it finds still a home. That section is written by Eduardo Licaèga, whom our Buffalo physicians will well remember as president of the American Public Health Association when it met here two years ago. On this account the article will possess an added interest.

The other articles treated of in this volume are plague, glanders, anthrax, foot-and-mouth disease, actinomycosis, rabies, pyemia and septicemia, all of which are written by experienced observers and most of whom are experts in the fields in which they discourse.

The volume is a continuation of the series that has already taken a high stand with the profession, and promises, when completed, to be the most complete reference encyclopedia of the practice of medicine extant. The publishers are making the work very attractive.

---

THE JOHNS HOPKINS HOSPITAL REPORTS. Report in Gynecology. Volume VII. Nos. 1 and 2. Baltimore: The Johns Hopkins Press. 1898.

This institution is somewhat famous for the publication of elaborate reports that, if they do not exhaust the subject, often do the reader. The report under consideration is published with a view, as the author undisguisedly asserts in its beginning, "to prove, from a review of 1,700 abdominal section cases performed from the opening of the Johns Hopkins Hospital in 1889 up to October 1, 1896, that not only is drainage valueless in the great majority of cases in which it has hitherto been used, and is still used by some surgeons and gynecologists, but that it is frequently productive of harm." This question has been so frequently discussed in the special medical societies of late years and so clearly settled as to practice, as well as theory, that we hardly see the necessity of an elaborately weighty report to prove what is already known and is practically unchallenged, or to disprove what every practical abdominal surgeon knows to be the truth.

---

HANDBOOK FOR THE HOSPITAL CORPS OF THE U. S. ARMY AND STATE MILITARY FORCES. By CHARLES SMART, Deputy Surgeon-General U. S. A. Approved by the Surgeon-General of the Army. Second revised edition. Duodecimo, 358 pages, illustrated. New York: William Wood & Co. 1898.

The usefulness of this handbook is testified to on all hands, and especially by the fact that those most familiar with it are loudest in its praise. It is written by one of the most accomplished officers in the medical staff of the army, not one of which is more familiar with the needs of the hospital corps. Dr. Smart has seen service in every capacity and all grades up to his present one, in both the volunteer army during the civil war, and in the regular army since that time. Moreover, he is a thorough student of the whole science of medicine as well as of military medicine, and could not fail to be interesting upon any subject which engaged his pen. His thorough knowledge of camp sanitation and military hygiene made him a most valuable aid to the Surgeon-General during the war with Spain, and this book has been of inestimable use to the officers and men of the war who have been so fortunate as to obtain it. It is a safe and adequate guide upon the subjects of which it treats and is commended to the medical officers of the national guard, as well as to those of the regular and volunteer forces in the service of the United States. Every military medical officer should become familiar with its teachings and every commanding officer of company and regiment should make it his duty to study it.

Diseases of the Stomach. By William W. Van Valzah, A. M., M. D., Professor of General Medicine and Diseases of the Digestive System in the New York Polyclinic Medical School and Hospital, and J. Douglas Nisbet, A. B., M. D., Adjunct Professor of General Medicine and Diseases of the Digestive System in the New York Polyclinic. Octavo volume of 674 pages. Illustrated. Philadelphia: W. B. Saunders, 925 Walnut street. 1898.

The stomach of late has been one of the best worked fields of the entire anatomy. Formerly this was not the case; it was even a neglected organ in so far as scientific investigation is concerned, physicians being content with accepting its subjective symptoms as indicating its pathology and treatment. Now, however, in keeping with the progress in other fields, the functional and organic disorders of the stomach have become about as well understood, and are as intelligently managed as are those of any organ—certainly as well as, or better than, any other organ in the digestive tract.

The work before us has the initial merit of simplicity. The author begins without preface to introduce the reader to his subject, and he hammers at it continually until he completes it. His introduction and classification are brief, next a short section on diagnostic methods, and then the subject proper. Van Valzah is especially strong in his chapter upon diet. There is no greater truth than that uttered on p. 168: "There is no general dietetic cure of the diseases of the stomach. Not only should the diet be suitable for the particular disease, but it should also be proper for the particular patient." It would be well for the laity and especially for our "diet cure" friends to ponder not only this axiomatic fact, but also much more that this author has to say on the subject.

The dynamic affections of the stomach are considered in the fourth section of the book. These are the least understood, or the most misunderstood, of all stomach affections. Our author is clear, forceful and instructive in regard to them. This section will repay careful study by even physicians most experienced in the diseases of the digestive tract.

The next section is devoted to the consideration of the anatomical diseases of the stomach, and is that part of the subject found of greatest interest to the general practitioner of medicine. Van Valzah is not only satisfying in his diagnosis, but is especially so on the subject of treatment. While he is liberal in citing the views of others, he is never doubtful as to his own plans. Finally, we come to the last section in which the vicious cycles of the stomach are dealt with. Every physician, no matter what his specialty, ought to read, even carefully study, this section of the book. It is full of mental pabulum, and will be sure to clear up questions of doubt, especially in the minds of those prone to overestimate the part the stomach itself plays in generating diseases of the system in general.

When we speak of Van Valzah we wish to include his associate, Dr. Nisbet, who is entitled to share full credit with his senior in this admirable treatise. It is written in clear, terse, sometimes epigrammatic English, and is to be regarded as one of the most practical of all the many good treatises on the stomach that have lately been put forth. We could wish that every physician would possess himself of the book and appeal to it on every doubtful occasion.

---

Brief Essays on Orthopedic Surgery. By Newton M. Shaeffer, M. D., Surgeon-in-Chief to the New York Orthopedic Dispensary and Hospital, etc. Duodecimo, pp. 81. New York: D. Appleton & Co. 1898.

This is a collection of papers published by the author in several medical journals, beginning January 26, 1884, and ending March 27, 1897. As they are accessible in the original places of their publication, and as they settle no moot question pertaining to this specialty, and further, as they present nothing new on this subject, we are unable to find any adequate reason for reprinting them in this separate form, attractive though it has been made by the publishers.

---

Text-Book of Medical Jurisprudence and Toxicology. By John J. Reese, M. D., late Professor of the Principles and Practice of Medical Jurisprudence in the University of Pennsylvania. Fifth edition, revised and enlarged by Henry Leffmann, M. D., Pathological Chemist, Jefferson Medical College Hospital, Chemist State Board of Health, Professor of Chemistry, Woman's Medical College of Pennsylvania, etc. 640 pages. Philadelphia: P. Blakiston's Son & Co. 1898.

The popularity of this text-book, written especially for students of legal medicine and containing the essentials of the science, is unquestioned. It is testified to most heartily in the demand foı successive editions at comparatively short intervals. The author was a teacher of the subject for more than twenty years before he put forth this book, and this gave him ample experience. The result has been that it has become the most sought for treatise of the handy variety in the English language. It takes a place somewhat below the large works in the estimation of the well-equipped advanced worker, who desires exhaustive reference volumes, but the practical student and every-day practitioner turns to this book with a sense of satisfaction not elsewhere obtained.

The editor has taken advantage of the preparation of this new edition to add new methods of material value and thus to keep it abreast of the progress of the science. We commend it as the best of its class and as all that is necessary for those for whom it is especially designed.

---

Public Health Reports (formerly Abstract of Sanitary Reports). Issued by the Supervising Surgeon-General, Marine Hospital Service. Volume XII., Nos. 1–53. Washington: Government Printing Office. 1898.

These reports are of great value to every person interested in public health and sanitation, as applied to the prevention of disease through state and municipal care. They contain a vast amount of information, and since they have been sent out bound in substantial form they have become easy of reference and their value is greatly increased. They can be obtained by public health officers upon application to the bureau at Washington that issues them.

---

TRANSACTIONS OF THE MEDICAL ASSOCIATION OF CENTRAL NEW YORK. Thirtieth annual meeting held at Buffalo, N. Y., October 19, 1897. Charles A. Van der Beek, M. D., Secretary. Volume IV. Buffalo Medical Journal Print. 1898.

This brochure of 145 pages is a record of the work of this association transacted in a single day. We know of no similar body that does as much and as creditable work in the same length of time, or that places it on record in better form. Since the association began the publication of its proceedings the scientific value of its work has increased in a marked degree. When another volume shall be published, the five then issued can be bound in substantial form and thereby a valuable book containing about 500 pages will be created. The forthcoming meeting of the association at Auburn is expected to be an unusually interesting one, hence the next volume promises to be fully up to its predecessors.

---

A SYSTEM OF PRACTICAL MEDICINE. By American Authors. Edited by ALFRED LEE LOOMIS, M. D., late Professor of Pathology and Practical Medicine in the New York University, and WILLIAM GILMAN THOMPSON, M. D., Professor of Medicine in the Cornell University Medical College, New York. In four imperial 8vo volumes. Vol. IV.—Diseases of the Nervous System and Mind; Vasomotor and Trophic Disorders; Diseases of the Muscles; Osteomalacia; Rachitis; Rheumatism; Arthritis; Gout; Lithemia; Obesity; Scurvy; Addison's Disease. 1,099 pages, 95 engravings and 8 full-page plates in colors and monochrome. For sale by subscription. Philadelphia and New York: Lea Brothers & Co., Publishers. 1898.

The fourth and last volume of this elaborate, interesting and instructive system of medicine presents for our consideration a most engaging group of diseases. First of these pertain to the nervous system and in the first section are presented diseases of the peripheral nerves, by Frederick G. Finley. Neuritis and facial neuralgia are the most attractive of these, because they are ever present in the practice of the family physician and from this work he may derive inspiration for their management.

Diseases of the spinal cord in their several subdivisions are next offered, and they are written by M. Allen Starr, Christian A. Herter, Edward D. Fisher and D. D. Stewart. They are somewhat exhaustively considered, and this portion of the work cannot fail to be attractive to neurologists. Diseases of the medulla and pons form a section by themselves and are dealt with by Christian A. Herter. Then comes diseases of the brain that are treated of in their severality by J. T. Eskridge, F. T. Miles, F. X. Dercum and Edward D. Fisher. These, too, are somewhat exhaustively considered and form an attractive portion of the work.

In the next section functional nervous disorders are presented to our view and these are discoursed upon in their several subdivisions by Frederick Peterson, Edward D. Fisher, Charles L. Dana, William H. Thompson, James J. Putnam, Charles K. Mills, Morton Prince, W. Gilman Thompson and Wharton Sinkler. These names give an earnest of the character of their work, and we need scarcely add that it is uniformly well done.

Diseases of the muscles, by Frederick G. Finley, are arranged in a separate section and then we come to a group of miscellaneous subjects that closes the volume. There are osteomalacia, by Finley; rachitis, by Jacobi; Addison's disease, by Coleman, and rheumatism, gonorrheal arthritis, arthritis deformans, gout, lithemia, obesity and scurvy, by Gilman Thompson.

From the foregoing synopsis of subjects and authors it will be observed that this volume is one of the most important of the series. Moreover, it has been prepared with great care and will commend itself to the specialist as well as to the practitioner of general medicine. Indeed, the neurologist that expects to keep pace with his specialty, and who as a matter of course must have *all* its literature —certainly its latest and best—will be compelled to possess himself of this volume.

It is handsomely made up uniformly with the other volumes of the series, which, now complete, forms one of the most comprehensive, scientific and instructive additions to the literature of general medicine that has been issued within recent years.

---

MODERN GYNECOLOGY. A Treatise on Diseases of Women. Comprising the results of the latest investigations and treatment in this branch of medical science. By CHARLES H. BUSHONG, M. D., Assistant Gynecologist and Pathologist to the Demilt Dispensary, New York; formerly Attending Physician to the Northern Dispensary, New York. Illustrated. Second edition, enlarged. New York: E. B. Treat & Co. 1898.

In the issue of the JOURNAL for December, 1893, Volume XXXIII., p. 305, we published some comments on the first edition of this work in which, while in the main were favorable to the book, we yet cited some points wherein we thought improvement might be made. Without repeating ourselves in detail at this time we may remark that our suggestions then made will, for the most part, apply now. This is especially true as regards some of the illustrations.

However, the volume betrays the work of a conscientious student, who is improving his treatise as time advances. He has remodeled some of its chapters, rewritten others and added a new one on hygiene and exercise. It is a handy and useful book of reference for the general physician, and will serve as a primary study for the beginner in this branch of medicine. The publishers have done all that can be done to make the book attractive in its mechanical make-up.

Tropical Diseases. A Manual of the Diseases of Warm Climates. By Patrick Manson, M. D., LL. D. (Aberd.), London. One volume of 652 pages, 12mo, illustrated by 88 wood engravings and 2 colored plates. New York: William Wood & Co. 1898.

In view of our prospective possessions in the tropics of both hemispheres it is opportune that we should have a manual of their diseases. Books in general on this subject have been too unwieldy for the traveler, but this one is convenient as to size and is excellent in its mechanical arrangement.

The fevers of the tropics possess unusual interest at this time, rendered doubly so by the fact that our returned and returning soldiers are quite generally affected, in greater or less degree, with some form of fever. Malarial and typhoid fevers appear to be the most prevalent, but, no doubt, there are other forms obscure in type or indistinct in expression that this volume will aid in elucidating.

Besides its handling of these subjects in a most intelligent and satisfactory manner, it sets forth with equally strong portrayal much of great interest in abdominal diseases, infective granulomatous diseases, animal parasites and their associated diseases, skin diseases and of local diseases, undetermined and uncertain nature.

Its numerous illustrations are greatly helpful to a completer understanding of many subjects dealt with, especially the skin and parasitic affections. It is a most commendable treatise, convenient in form, and should be at hand for consultation by every physician who visits the tropics or has the care of those who are returning therefrom.

---

Medical and Surgical Report of the Presbyterian Hospital in the City of New York. Edited by Andrew J. McCosh, M. D., and Walter B. James, M. D. Volume III., pp. xv.—414. January, 1898.

The reports of this hospital are among the very best sent out by similar institutions in this country. The report before us is no exception to the rule. In addition to the usual preliminary pages, containing the lists of managers and staff, it consists of 414 pages, small quarto in size and beautifully tinted, devoted to scientific work. The first paper is by Wm. H. Draper, entitled, On some of the collateral functions of a hospital. It is a most interesting setting forth of the way in which a hospital may be made most useful as a means of instruction, and thus become of greater value to its patients as a place in which to be cured or relieved of disease. Dr. Draper is always an interesting writer, and in this instance has invaded a field of peculiar attractiveness—one not heretofore ploughed and harrowed.

The book is filled with reports of unusual cases and details of their management—not with simply brilliant surgical operations, that of necessity interest the few.

This report may well serve as a model; it is not filled with tables and figures, as is usual in hospital reports, but with science. It is a credit to its editors and to the whole staff of the Presbyterian Hospital.

---

ATLAS OF LEGAL MEDICINE. By Dr. E. v. HOFMANN, Professor of Legal Medicine and Director of the Medico-Legal Institute at Vienna. Authorised translation from the German. Edited by Frederic Peterson, M. D., assisted by Aloysius O. J. Kelly, M. D. Fifty-six plates in colors and 193 illustrations in black. Philadelphia: W. B. Saunders. 1898.

It would be impossible to overstate the importance of this work, because it furnishes aid in a field heretofore but little cultivated. Medical jurisprudence has usually been meagerly illustrated and for the most part by indifferent or almost valueless cuts. This atlas can be referred to in the reading of any work on legal medicine that has ever been published and especially will it be recognised of inestimable value to coroners and those engaged at inquests. The accuracy of the pictures and the clearness with which they are described make the work of great moment and practically disarm criticism.

---

REPORT OF THE HEALTH DEPARTMENT OF THE CITY AND COUNTY OF SAN FRANCISCO for the Fiscal Year ending June 30, 1897. Edmond Godchaux, Secretary, San Francisco. 1897.

The importance of complete annual reports of the work of municipal health boards becomes more and more apparent as the years progress. This one bespeaks the energy and efficiency of public municipal sanitation on the Pacific coast. It contains 463 octavo pages, quite largely made up of tables. It is, nevertheless, a contribution to the cause of preventive medicine, and is a credit to the San Francisco health authorities.

